# Effects of Ethnicity and Spiritual Intelligence in the Relationship Between Awe and Life Satisfaction Among Chinese Primary School Teachers

**DOI:** 10.3389/fpsyg.2021.673832

**Published:** 2021-07-12

**Authors:** Zhenhui Liu, Xin Li, Tonglin Jin, Qianguo Xiao, Tena Wuyun

**Affiliations:** ^1^School of Psychology, Inner Mongolia Normal University, Hohhot, China; ^2^School of Teacher Education, Honghe University, Mengzi, China; ^3^Laboratory of Cognition and Mental Health, Chongqing University of Arts and Sciences, Chongqing, China

**Keywords:** awe, spiritual intelligence, life satisfaction, ethnicity, primary school teacher

## Abstract

Based on the broaden-and-build theory of positive emotions, this study explored the mediating effect of spiritual intelligence between awe and life satisfaction among Chinese primary school teachers and whether this effect was moderated by ethnicity. Participants comprised 569 teachers from 24 primary schools in southwestern China, where many of the ethnic minority groups of China reside. Awe and spiritual intelligence were found to positively predict life satisfaction among primary school teachers, while awe also indirectly influenced life satisfaction through the partial mediation of spiritual intelligence. Ethnicity was also found to moderate the relation between awe and life satisfaction, i.e., when compared with the Han teachers, there is a more significant and positive relation between awe and life satisfaction in ethnic minority teachers. These findings not only indicate the critical role of awe in promoting life satisfaction of primary school teachers but also especially show that awe embodied in the traditional cultural activities makes it easier to breed life satisfaction in ethnic minority teachers.

## Introduction

The “Guidelines for Mental Health Education in Primary and Secondary Schools” promulgated by the Ministry of Education of the People’s Republic of China in 2012 clearly states that “Teachers’ mental health education should be regarded as an important aspect of teachers’ education and career development.” In target tasks of Opinions on Comprehensively Deepening the Construction and Reform of the Faculty in the New Era (2018), the State Council of China proposed that “respecting teachers and attaching great importance to education has become a common trend, so that the majority of teachers have a sense of happiness in their posts, a sense of accomplishment in their careers, and a sense of honor in society.” How to enhance the physical and mental health and happiness of teachers has become an important issue in the field of education research. The study found that improving the salary of teachers and understanding the meaning of work, family companionship, and harmonious interpersonal relationship were the appeals of primary and secondary school teachers in ethnic minority areas to improve their wellbeing, while developing their happiness ability (i.e., happiness is a kind of ability) became the key challenge (Deng and Cheng, 2009; Yang and Jing, 2019). Hence, we thought that the physical and mental health and happiness of teachers could be facilitated by cultivating awe in the process of teacher education.

Awe is an organic combination of the need for adaptation, fear, enlightenment, reverence, humility, and other emotions in individuals when they encounter something beyond their cognitive structure or original frame of reference in a given dimension or domain (Keltner and Haidt, 2003). Awe can induce the distinctive effects of individuals, such as the small self, humility, mood improvement, and prosocial value orientation (Dong et al., 2013). Some studies indicated that awe has positive facilitation on the physical and mental health and subjective wellbeing of people (Rudd et al., 2012; Krause and Hayward, 2015; Stellar et al., 2015; Dong and Ni, 2019; Zhao et al., 2019). For instance, higher dispositional awe is associated with higher resilience, in general, and predicts lower negative health effects (Tian et al., 2016; Koh et al., 2017; Atamba, 2019). Anderson et al. (2018) recently found that, compared with general positive emotion, awe experience induced by outdoor activities (e.g., rapid rafting) was able to give predictions on the changes of individual happiness and stress-related symptoms a week later, and awe experience plays a positive role on improvement in wellbeing. Life satisfaction could be taken as a reactive indicator to assess the mental health and happiness of teachers (Greenspoon and Sasklofske, 2001). Based on the perspective of positive psychology, the main purpose of this study is to explore the possible psychological mechanism of awe affecting life satisfaction among primary school teachers.

## The Relationship Between Awe and Life Satisfaction

Life satisfaction is the subjective evaluation of quality of life of an individual based on an intrinsic value standard, which is also the cognitive component of subjective wellbeing (Diener et al., 1985). Subjective wellbeing is the overall evaluation of the quality of life by individuals based on their positive and negative emotional experiences and life satisfaction. It is believed that life satisfaction increased by positive emotion but decreased by negative emotion (Dong and Zhang, 2013). As a positive emotion of self-transcendence, awe can reconstruct and expand the cognitive structure of an individual, making it easier for an individual to obtain rich and long-term positive emotions (Keltner and Haidt, 2003). One of the studies induced the experiences of happiness, calmness, and awe through two different methods, namely, a video program and a reading task. It was found that individuals who have experienced awe tend to choose products that offer a spiritual experience, and awe can offset the negative effects of materialism and enable people to actively face life and pursue the spiritual life, and it also implies a higher level of life satisfaction (Rudd et al., 2012; Van Cappellen and Saroglou, 2012; Ye and Dong, 2015).

According to the broaden-and-build theory of positive emotions (Fredrickson, 1998, 2001), awe, as a kind of self-transcendent positive emotion, can broaden the thoughts and behaviors of an individual and can build long-term physical and mental resources. Awe can increase the psychological capital of an individual and play a direct role in the subjective wellbeing of primary school teachers. It can also improve the subjective wellbeing by alleviating the negative impact of occupational stress (Cohn et al., 2009; Zhang et al., 2014; Tian et al., 2016; Atamba, 2019). In addition, the experience of awe is so vast that it leads people to recognize small self and humility, thereby leading to more altruistic behavior, healthier social and interpersonal relationships (e.g., increased ethical decision-making and more generosity to a stranger) (Piff et al., 2015; Stellar et al., 2017), and higher levels of mental resilience and wellbeing (Krause et al., 2016; Tian et al., 2016).

## The Mediating Role of Spiritual Intelligence Between Awe and Life Satisfaction

Spiritual intelligence was defined as the ability to apply, manifest, and embody spiritual resources, values, and qualities to enhance daily functioning and wellbeing (Amram and Dryer, 2008). A key function of spiritual intelligence was to solve problems of meaning and value in life (Adams and Hyde, 2008). By definition, awe and spiritual intelligence are closely related to the meaning of life, adaptive function, spiritual resources, and connectedness with the world around them (Emmons, 2000; Wolman, 2001; King and DeCicco, 2009; Hosseini et al., 2010). Existing studies have provided arguments from different aspects. For example, dispositional awe is significantly and positively correlated with the meaning of life (Zhao et al., 2019). Awe experience makes people feel connected to others and the environment (Yaden et al., 2018). Awe implies an open and flexible mindset that enables people to solve problems creatively (Isen et al., 1987; Chirico et al., 2018). Thus, awe may be directly correlated with spiritual intelligence, as the former can predict the latter.

It is well known that the intelligence of an individual is to some extent influenced by external circumstances, such as ethnic culture and religion. According to this logic, spiritual intelligence is assumed to be influenced by the psychosocial and emotional experiences of a person, for example, a person who has a diverse background should have higher spiritual intelligence (Emmons, 2000). People with high spiritual intelligence were more tolerant in dealing with stresses in life and were also better able to adapt to situations (Smith, 2004). Spiritual intelligence not only involves the capacity for being but also exists as a set of mental capacities distinct from behavioral characters and experiences, which meets the established standards of intelligence (Gardner, 1983; Sternberg, 1997). Esmaili et al. (2014) proposed that compatibility with the events and experiences of life and conscious expansion were the main factors contributing to the growth of spiritual intelligence. According to the existing theories and studies, awe could expand the internal psychological resources and cognitive perspective of an individual (Fredrickson, 2001; Keltner and Haidt, 2003). Shiota (2014) believed that awe could promote the ability to form and update the abstract mental representation of the world, improve the degree of processing new information from the environment, and enhance cognitive flexibility. Consistent with this, Lin et al. (2020) proposed that dispositional awe is closely related to the Zhong-Yong thinking, which enables people to understand the world from a global perspective and thus obtain a kind of balance. Therefore, awe was conducive to the development of spiritual intelligence.

In general, the development of spiritual intelligence can be seen as the use of abilities and spiritual resources in the actual situation. There is a two-way promoting effect between awe and spirituality (Saroglou et al., 2008; Van Cappellen and Saroglou, 2012; Preston and Shin, 2016). However, spiritual experiences do not affect intellectual humility and the need for cognitive closure, both of which are associated with the epistemological aspects of awe (Preston and Shin, 2016). Awe can theoretically promote spiritual experience and thus promote the spiritual intelligence of an individual (Green and Noble, 2010). A study by Bonner (2015) shows that awe could positively predict spiritual intelligence, and it indicates the possibility that awe could improve spiritual intelligence. Vaughan (2002) believed that spiritual intelligence is related to awe in two ways as follows: (1) spiritual intelligence is usually related to the improvement of aesthetic sensitivity and (2) spiritual intelligence helps to explore existing uncertainty and reduces the demand for certainty. The views of Vaughan (2002) have been verified to varying degrees. Researchers have shown that the profound aesthetic experience of spatial pictures and music is often associated with the experience of awe. Openness can predict the level of awe in the aesthetic process. Openness, such as the experience of awe, can expand the way people think about themselves and the world (Silvia et al., 2015). In contrast, dispositional awe is negatively correlated with the need for cognitive closure, and high dispositional awe means high tolerance for uncertainty (Shiota et al., 2007). For example, awe experiences are associated with greater positive emotions while waiting for uncertain information and, to a lesser extent, with lower negative emotions and anxiety (Rankin et al., 2019).

Spiritual intelligence emphasizes the adaptive function of spirituality for individuals, requires multiple cognitive modes, and combines the inner spirit and mental state with the outer life (Vaughan, 2002). Previous research shows that spiritual intelligence is a positive and significant predictor of general health and wellbeing (Amirian and Fazilat-Pour, 2016), and it positively affects the job satisfaction and life satisfaction of an individual (Chen, 2013; Kaur, 2013; Benedict-Montgomery, 2014; Marashi et al., 2016). Therefore, this study intends to explore the mediating effect of spiritual intelligence between awe and life satisfaction among primary school teachers.

## The Moderating Role of National Attributes Between Awe and Life Satisfaction

In different cultures (e.g., collectivist and individualistic), individuals experience awe differently. The differences in the inducing source, frequency, and content of awe are related to cultural values (Bai et al., 2017). Han (2017) found that Mongolian cultural aspects such as customs, ceremonies, and concepts demonstrated a cultural particularity when evoking awe. For Mongolian college students, the awe level aroused by national images (i.e., many traditional festivals in Mongolia, ritual activities such as Oboo festival, Fire festival, Grassland God or Mongke Tengri festival, and Naadam) is higher than that aroused by natural images. In Mongolian college students who have lived in the pastoral areas for a long time, the awe level aroused by national images is even higher than that in the Mongolian college students who have lived in the pastoral areas for a short time, and, in addition, awakening awe made participants more self-disciplined, virtuous, moral, and satisfied with their life. Therefore, different national cultures have different influences on awe and awe-related behaviors, and national traditional culture and environment can positively predict individual awe levels.

Awe is an emotional response by people of low social rank to people of high social rank, whereby they place their own interests in a subordinate position to integrate into the social hierarchy of the group to which they belong and to adapt to the needs of different relationships and their cultural backgrounds (Keltner and Haidt, 2003; Goetz and Keltner, 2008). Many awe triggers, such as spiritual leaders, art, religious beliefs, and major social ritual, are central to the meaning system and collective identity of a culture (Van Cappellen and Saroglou, 2012; Van Cappellen and Rimé, 2014; Han, 2017). Awe could be a perception of threat and danger, and it could also (more often) occur in most areas such as nature, social events, and religion (Gordon et al., 2016). Preston and Shin (2016) found that people with religious beliefs are more inclined to take religious activities, life and death events, as the source of spirituality, and spiritual experience induces their feelings of awe. For example, the awe of God makes people feel deeply connected with others, which make them more satisfied with their life (Krause and Hayward, 2015). In contrast, religious beliefs have a positive effect on mental health, and ethnic attribute plays a moderating role in this relationship (Tabak and Mickelson, 2009). According to the religious prosociality hypothesis (Dong et al., 2015), high religious belief to some extent contributes to the psychological wellbeing of people.

In addition, several studies have found that ethnic culture and identity had a positive predictive effect on the life satisfaction of peoples and that the life satisfaction of ethnic minorities is higher than that of non-ethnic minorities such as the Han (Zhong et al., 2007; Shu, 2008; Hu, 2009; Li et al., 2014; Zhao and Mao, 2014). One possible explanation is that, compared with the Han nationality, the ethnic original culture is less impacted by modernization (Yin, 2005). To some extent, the diversity and unity pattern of the Chinese nation since ancient times has ensured this (Fei, 1999). Many minority communities, localized in southwestern China, still maintain the independence and integrity of traditional culture and “Harmony without uniformity” cultural values to some extent (Liu and Wuyuntena., 2017). That is, according to the concept of Bicultural Identity Integration, different cultures could be independent and compatible (Benet-Martínez et al., 2002), for example, “in my life, I can keep Tibetan and Chinese cultures independent of each other” (Yang et al., 2015). In the process of integrating different cultural identities, individuals with a harmonious cultural orientation are more likely to be psychologically well adapted and satisfied with their lives (Mok et al., 2007; Yang et al., 2015; Hu et al., 2016). Ethnic minority teenagers have consisten ethnic cultural identity (Hu et al., 2014). Therefore, from the perspectives of culture and collective identity, awe that is embodied in traditional culture can partially explain the ethnic differences in life satisfaction among primary school teachers. We propose that ethnic attributes have a moderating effect on the relation between awe and life satisfaction of primary school teachers.

## Research Hypotheses

Thus, guided by the relevant theories and the results of existing research as the foregoing, this study examines the role of awe and spiritual intelligence on life satisfaction among primary school teachers, testing the following three hypotheses:

**Hypothesis 1:** Awe has a positive predictive effect on spiritual intelligence and life satisfaction among primary school teachers.
**Hypothesis 2:** Spiritual intelligence mediates the relation between awe and life satisfaction of primary school teachers.
**Hypothesis 3:** Ethnicity moderates the relation between awe and life satisfaction of primary school teachers.

## Materials and Methods

### Participants and Procedure

In this study, graduates engaged in basic education were recruited through the WeChat group of normal college graduates. A total of 30 graduates (i.e., teachers currently engaged in basic education) were randomly selected, among which 24 graduates were willing to cooperate with the researcher to carry out this survey in their own primary schools. The voluntary principle was adopted to recruit their colleagues to participate in this survey (i.e., offline paper questionnaires). Since they belong to different primary schools, 24 graduates meant 24 primary schools.

A total of 658 teachers from 24 primary schools in Yunnan Province, China were selected using the convenience and random sampling method. The survey data were obtained through written questionnaires and network measurement. Voluntary participation and anonymity were emphasized. We entrusted local primary teachers to issue and collect the questionnaires. A total of 569 teachers returned completed questionnaires, representing an effective response rate of 86.5%. Of the final teacher sample, 166 (29.2%) were men and 403 (70.8%) were women; 355 (62.4%) were Han people and 214 (37.6%) were from ethnic minorities (i.e., Miao, Tu, Wa, Yi, Hani, Jingpo, Dai, Naxi, Bai, Zhuang, Lahu, Lisu, Hui, and Yao); 126 were in tertiary duty in primary school education (22.1%), 251 were in secondary duty (44.1%), and 192 were in primary duty (33.7%); and 134 had a junior college degree (23.6%) and 435 had a bachelor’s degree (76.4%). This distribution covers all the prefecture-level cities in Yunnan Province and represents the distribution characteristics of primary school teachers in the southwest minority areas.

This study was carried out in accordance with the recommendations of gridlines by the Ethics Committee of Honghe University (Yunnan Province) in China. All subjects provided written informed consent.

### Measures

#### Awe

The subscale of awe is from the 38-item Dispositional Positive Emotion Scale (Shiota et al., 2006), which is a self-report instrument with seven dispositional positive emotion subscales, namely, awe, joy, compassion, love, contentment, pride, and amusement.

The subscale of awe includes six items (e.g., “I often feel awe.”). Respondents answered these items on a 7-point Likert-type scale ranging from 1 = “strongly disagree” to 7 = “strongly agree.” The confirmatory factor analysis was performed using Amos software version 21.0, and the fit indexes were χ^2^/*df* = 4.76, NFI = 0.94, TLI = 0.92, CFI = 0.97, RMSEA = 0.08. The Cronbach’s alpha for the awe subscales was 0.78.

#### Spiritual Intelligence

The 28-item Spiritual Intelligence Scale was used to assess the spiritual intelligence of participants (Chen, 2013). This version was from the Integrated Spiritual Intelligence Scale (Amram and Dryer, 2008) and was further revised in Chinese by Chen (2013). It consists of five dimensions, namely, consciousness (e.g., “During an activity or conversation, I monitor and notice my thoughts and emotions”), grace (e.g., “My life is a gift, and I try to make the most of each moment”), meaning (e.g., “I derive meaning from the pain and suffering in my life”), transcendence (e.g., “I am aware of a wise- or higher-self in me that I listen to for guidance”), and presence (e.g., “I tend to think about the future or the past without attending to the present moment”). Respondents answered these items on a 6-point Likert-type scale ranging from 1 = “not at all” to 6 = “very much so.” The fit indexes were χ^2^/*df* = 3.22, NFI = 0.95, TLI = 0.94, CFI = 0.96, RMSEA = 0.06. The Cronbach’s alpha for the Integrated Spiritual Intelligence Scale was 0.89, and the coefficients of each dimension ranged from 0.77 to 0.86.

#### Life Satisfaction

Life satisfaction was assessed using the Satisfaction with Life Scale (SWLS) compiled by Diener et al. (1985). The SWLS is a 5-item scale, with items such as “My living conditions are very good.” Respondents answered these items on a 7-point Likert-type scale ranging from 1 = “strongly disagree” to 7 = “strongly agree.” The fit indexes were χ^2^/*df* = 2.37, NFI = 0.98, TLI = 0.98, CFI = 0.99, RMSEA = 0.05. The Cronbach’s alpha for the SWLS was 0.84.

## Results

### Control and Test of Common Method Bias

Program controls, such as anonymous protection, random order of scales, and various response formats (6- and 7-point scales), at 24 schools separated in time and space, were employed during data collection to decrease the common method bias effect as possible.

Following data collection, the Harman’s single-factor test (Podsakoff et al., 2003) was conducted to examine the common method bias. Seven factors were found to have eigenvalues greater than 1, and the first factor accounted for 25.19% of variance (i.e., below the critical standard of 40%). Moreover, we controlled the unmeasured latent method construct and compared the fit indices of three-, two-, and one-factor models. The results of the confirmatory factor analysis showed that the fit indexes of three-factor model (i.e., awe, spiritual intelligence, and life satisfaction) were χ^2^/*df* = 4.07, NFI = 0.91, TLI = 0.90, CFI = 0.92, RMSEA = 0.07. The fit indexes of the first two-factor model (i.e., awe + spiritual intelligence and life satisfaction) were χ^2^/*df* = 6.84, NFI = 0.79, TLI = 0.78, CFI = 0.81, RMSEA = 0.11, the second two-factor model (i.e., awe + life satisfaction and spiritual intelligence) were χ^2^/*df* = 11.59, NFI = 0.65, TLI = 0.61, CFI = 0.67, RMSEA = 0.13, and the third two-factor model (i.e., awe and spiritual intelligence + life satisfaction) were χ^2^/*df* = 13.12, NFI = 0.60, TLI = 0.55, CFI = 0.62, RMSEA = 0.15. The fit indexes of one-factor model (i.e., awe + spiritual intelligence + life satisfaction) were χ^2^/*df* = 15.83, NFI = 0.51, TLI = 0.45, CFI = 0.53, RMSEA = 0.16. This indicated that there was not a substantial amount of common method variance in this research, and the three-factor model was a good fit.

### Descriptive and Correlation Statistics

Table 1 presents the mean values, SDs, and demographic differences in dispositional awe, spiritual intelligence, and life satisfaction. The results show that the life satisfaction of teachers with CDB was significantly lower than that of teachers with BDA (*p* < 0.05). There were significant differences in spiritual intelligence and life satisfaction among teachers with different professional titles (all *p* < 0.05), and the spiritual intelligence and life satisfaction of teachers with a third-grade title were higher than that of the other two groups. The working location significantly affects the dispositional awe (*p* < 0.01), spiritual intelligence (*p* < 0.05), and life satisfaction (*p* < 0.01) of teachers, and teachers who worked in the county had the highest dispositional awe, life satisfaction, and spiritual intelligence. Age was significantly and positively correlated with dispositional awe (*r* = 0.09, *p* < 0.05), spiritual intelligence (*r* = 0.20, *p* < 0.01), and life satisfaction (*r* = 0.22, *p* < 0.01).

**TABLE 1:** Demographic differences in awe, spiritual intelligence, and life satisfaction.

**Variables**	**Awe**			**Spiritual intelligence**			**Life satisfaction**		
	***M* (*SD*)**	***F***	**η^2^*_*p*_***	***M* (*SD*)**	***F***	**η^2^*_*p*_***	***M* (*SD*)**	***F***	**η^2^*_*p*_***
Gender	Male 4.02 (0.86); Female 3.92 (0.91)	1.44	0.00	Male 4.02 (0.56); Female 4.00 (0.55)	0.16	0.00	Male 3.75 (1.30); Female 3.76 (1.29)	0.01	0.00
Education	CDB 4.03 (0.93); BDA 3.92 (0.89)	1.67	0.01	CDB 4.11 (0.53); ABDA 3.97 (0.56)	2.71	0.01	CDB 4.00 (1.21) > BDA 3.68 (1.31)	3.69*	0.02
Ethnicity	Em 3.95 (0.94); Han 3.94 (0.87)	0.01	0.00	Em 4.03 (0.61); Han 3.98 (0.52)	0.72	0.00	Em 3.89 (1.27) > Han 3.67 (1.30)	3.17	0.01
Professional title	Pf 3.93 (0.91); Ps 3.95 (0.86); Pt 3.97 (0.95)	0.10	0.00	Pf 3.94 (0.56) or Ps 3.98 (0.54) < Pt 4.17 (0.55)	6.71**	0.03	Pf 3.57 (1.23) or Ps 3.67 (1.29) < Pt 4.19 (1.30)	9.88**	0.04
Working Location	County 4.20 (0.80) > Country 3.98 (0.91) or Towns 3.87 (0.88) > Plc 3.66 (0.98)	4.24**	0.02	Towns 4.03(0.59); Plc 4.03(0.49); Country 3.93(0.53) < County 4.12(0.54)	2.63*	0.01	County 4.14 (1.30) > Country 3.56 (1.21) or Plc 3.54 (1.25); Towns 3.81 (1.36) > Country 3.56 (1.21)	4.57**	0.03
Age	*r* = 0.09*			*r* = 0.20**			*r* = 0.22**		

*p < 0.05, **p < 0.01. CDB, College degree and below; BDA, Bachelor degree or above; Em, Ethnic minorities; Pf, Ps, Pt, Primary school teachers with a first, second, third grade title; Plc, Prefecture-level cities.

Table 2 presents the means, SDs, and correlations among all the variables. The results show that awe and life satisfaction were significantly positively correlated (*r* = 0.23, *p* < 0.01), awe and spiritual intelligence were significantly positively correlated (*r* = 0.53, *p* < 0.01), and awe was positively correlated with its four dimensions, namely, consciousness, grace, meaning, and transcendence (*r* = 0.38–0.49, all *p* < 0.01), but the correlation coefficient between awe and presence dimensions was not significant (*r* = 0.08, *p* > 0.05). There was a significant positive correlation between spiritual intelligence and life satisfaction (*r* = 0.19, *p* < 0.01), and the four dimensions of mental intelligence, namely, consciousness, gift, meaning, and transcendence, were positively correlated with life satisfaction (*r* = 0.15–0.27, all *p* < 0.01), but presence dimensions negatively correlated with life satisfaction (*r* = −0.18, *p* < 0.01). These results furnished preliminary support for the hypothesized relationships.

**TABLE 2:** Means, standard deviations, and correlation coefficients of variables.

**Variables**	***M* ± *SD***	**1**	**2**	**3**	**4**	**5**	**6**	**7**	**8**
1.Awe	3.95 ± 0.90	1							
2.Consciousness	4.13 ± 0.76	0.38**	1						
3.Gift	4.29 ± 0.77	0.49**	0.62**	1					
4.Meaning	4.18 ± 0.87	0.40**	0.50**	0.64**	1				
5.Transcendence	3.85 ± 0.88	0.45**	0.47**	0.57**	0.64**	1			
6.Prensence	3.55 ± 0.84	0.08	–0.04	–0.06	–0.05	0.13**	1		
7. Spiritual intelligence	14.03 ± 1.95	0.53**	0.74**	0.84**	0.77**	0.80**	0.28**	1	
8. Life satisfaction	3.75 ± 1.30	0.23**	0.16**	0.25**	0.27**	0.15**	−0.18**	0.19**	1

**p < 0.01.

### Hypotheses Testing

As per the hypothesis model, the structural equation models in Amos 21.0 were used to test the mediation of spiritual intelligence (i.e., Han or ethnic minorities). After standardizing the variables awe, spiritual intelligence, and life satisfaction, the non-parametric percentile bootstrap method with deviation correction was adopted to test the mediating and moderating effects, with a bootstrapping of 2,000 resampling iterations (Wen and Ye, 2014). The testing processes were as follows:

Step 1. The results demonstrated that the direct paths of awe (β = 0.23, *p* < 0.01) toward life satisfaction were significant, confirming Hypothesis 1. The results will be helpful for further testing the mechanism of mediation.

**FIGURE 1:**
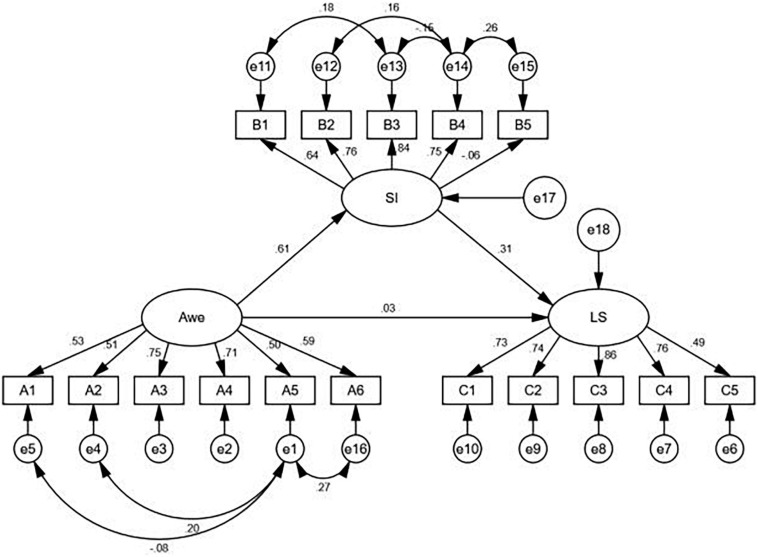
The mediating role of spiritual intelligence between awe and life satisfaction. B1-B5, consciousness, meaning, grace, transcendence, presence; SI, spiritual intelligence; LS, life satisfaction.

As shown in Figure 1 and Table 3, after adding the mediator spiritual intelligence, the fit indices were χ^2^/*df* = 3.12, NFI = 0.91, TLI = 0.92, CFI = 0.94, RMSEA = 0.06, indicating that the model was a good fit. Spiritual intelligence was a significant mediator (i.e., indirect effect = 0.19), and the 95% CI for the indirect effect (0.12, 0.28) did not include zero. The direct path of awe was not significant (β = 0.03, *p* > 0.05), and the indirect paths from awe to spiritual intelligence (β = 0.61, *p* < 0.001) and from spiritual intelligence to life satisfaction (β = 0.31, *p* < 0.001) were both significant. Therefore, spiritual intelligence plays a completely mediating role between awe and life satisfaction. Thus, Hypothesis 2 was supported.

**TABLE 3:** Path coefficient results for awe, spiritual intelligence, and life satisfaction.

**Variable**	**Estimate**	***SE***	***t***
Awe — > Spiritual intelligence(a)	0.61	0.05	7.82***
Spiritual intelligence — > Life satisfaction(b)	0.31	0.14	4.20***
Awe — > Life satisfaction(c’)	0.03	0.09	0.43

***p < 0.001.

**FIGURE 2:**
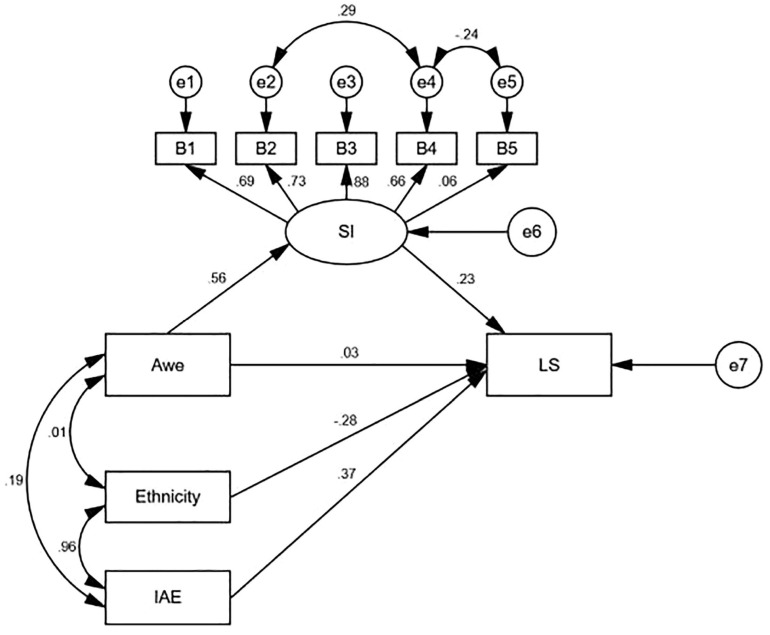
The internal mechanism model of awe affecting life satisfaction. B1-B5, consciousness, meaning, grace, transcendence, presence; SI, spiritual intelligence; LS, life satisfaction; IAE, the Interaction of Awe and Ethnicity.

Step 2. As shown in Figure 2 and Table 4, after adding the moderator ethnicity, the fit indices were χ^2^/*df* = 2.62, NFI = 0.98, TLI = 0.98, CFI = 0.99, RMSEA = 0.05, indicating that the model was a good fit. Spiritual intelligence was a significant mediator (i.e., indirect effect = 0.13), and the 95% CI for the indirect effect (0.07, 0.19) did not include zero. The direct path of awe was not significant (β = 0.03, *p* > 0.05), and the indirect paths from awe to spiritual intelligence (β = 0.56, *p* < 0.001) and from spiritual intelligence to life satisfaction (β = 0.23, *p* < 0.001) were both significant. In addition, the path from ethnicity to life satisfaction was not significant (β = −0.28, *p* > 0.05) and from awe to life satisfaction was significant (β = 0.37, *p* < 0.05). Therefore, spiritual intelligence plays a completely mediating role between awe and life satisfaction, and ethnicity was a moderator in the relationship of awe and life satisfaction.

**TABLE 4:** Path coefficient results for awe, ethnicity, spiritual intelligence, and life satisfaction.

**Variable**	**Estimate**	***SE***	***t***
Awe — > Spiritual intelligence(a)	0.56	0.02	11.86***
Spiritual intelligence — > Life satisfaction(b)	0.23	0.14	4.04***
Awe — > Life satisfaction(c1’)	0.03	0.07	0.66
Ethnicity — > Life satisfaction(c2’)	−0.28	2.40	–1.55
IAE — > Life satisfaction(c3’)	0.37	0.10	2.02*

*p < 0.05, ***p < 0.001. IAE, The interaction of Awe and Ethnicity.

**FIGURE 3:**
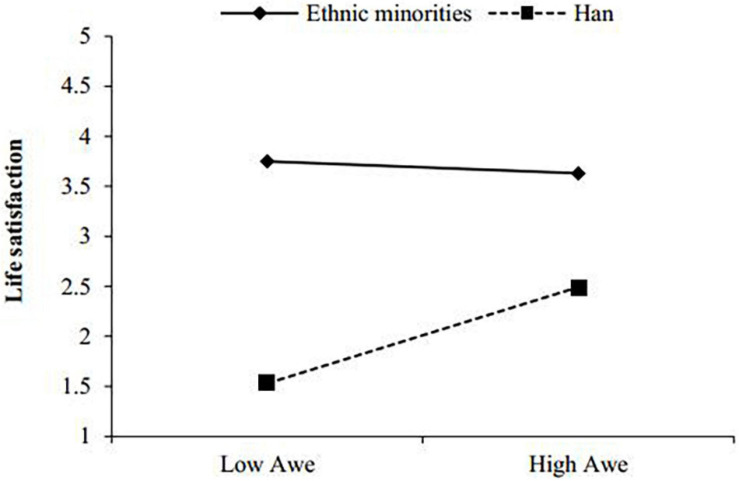
The Moderating Effect of Ethnicity on the Relationship between awe and life satisfaction.

The simple slope test (Figure 3) shows that awe had a significant predictive effect on life satisfaction for ethnic minority teachers, β = 0.47, *p* < 0.001, 95% CI: (0.25, 0.67), and awe also had a significant predictive effect on life satisfaction for Han teachers, β = 0.21, *p* < 0.01, 95% CI: (0.05, 0.37).

## Discussion

### Awe Positively Predicts Life Satisfaction

The results of this study show that awe in primary school teachers can positively predict life satisfaction: the higher the degree and frequency of awe experience, the higher the life satisfaction of primary school teachers will be. This finding is consistent with previous studies (Rudd et al., 2012; Dong and Ni, 2019). We believed that cognitive transformation is one of the important effects of awe. Research suggests that dispositional awe tends to be positively correlated with openness and extroversion (Shiota et al., 2006; Dong and Ni, 2019) and negatively correlated with lower need for cognitive closure (Shiota et al., 2007). Awe enables people to deal with a new stimulus with openness and a non-evaluative mindset, which is an important factor in the life satisfaction of primary school teachers (Le, 2011; Danvers and Shiota, 2017). Awe experience is a kind of self-transcendence, rather than self-oriented emotional arousal, which has the function of actively expanding or transforming cognitive perspective and can produce a sense of connectedness, accommodation, and sacredness with a larger existence. Hence, it may affect the cognitive assessment of life satisfaction of primary school teachers. Dispositional awe is negatively correlated with the importance of material wealth and its acquisition in the life of people and positively correlated with the meaning of life (Zhao et al., 2019). A previous study has shown that individuals tend to have lower wellbeing when they attach more importance to the sense of acquisition and improvement of material or resources (Kasser et al., 2014).

Positive psychology researchers proposed that awe, as a self-transcendent emotion, may promote individual wellbeing and personal change (Haidt and Keltner, 2004), and awe experiences in daily life and in the laboratory led to greater momentary wellbeing (i.e., compared with no awe experience) (Gordon et al., 2016). Awe could also reduce the negative impact of daily stress and negative emotions and improve life satisfaction (Rudd et al., 2012; Bai et al., 2021). Therefore, as a positive emotion of self-transcendence, awe has a distinct emotional effect, which promotes the growth and transformation of primary school teachers and improves their life satisfaction. However, it may consume more positive resources and energy to compensate for or to offset the negative effects caused by negative events and experiences, which leads to a weak association.

### The Mediating Role of Spiritual Intelligence

The results reveal a significant positive correlation between awe, spiritual intelligence, and life satisfaction with each other. Spiritual intelligence plays a completely mediating role in the relation between awe and life satisfaction. This result reveals that the higher the awe level of primary school teachers, the higher their level of spiritual intelligence, leading to an improvement in life satisfaction.

Awe derived from nature and the birth of new life surpasses the original frame of reference of peoples and generates the need for adaptation, so they achieve the balance between the internal and external environment. The experience of awe can enlighten and develop the mind. Zhu (2005) believes that awe is a kind of moral emotion with the educational value of being able to reach the soul and develop the spirit. Spirituality can be awakened and developed through awe at the wonder of life, nature, and the universe (Saroglou et al., 2008; Van Cappellen et al., 2013; Ye and Dong, 2015), and spiritual experience can promote the development of spiritual intelligence (Vaughan, 2002; Green and Noble, 2010). This finding that awe enables change in the mental model of primary school teachers is consistent with previous studies. Awe can positively predict spiritual intelligence (Bonner, 2015). Spiritual intelligence is the ability to find meaning, purpose, and value in life, connecting our actions and lives to a wider and richer context of giving meaning (Wolman, 2001; Alex and Ajawani, 2011). The shocking awe experience may be just the positive expansion and fit with the need of an individual for this kind of meaning-seeking (Cohen and Cairns, 2012; Zhao et al., 2019).

Spiritual intelligence is adaptive, enabling people to solve problems and achieve goals (Amram and Dryer, 2008; Hosseini et al., 2010), and the achievement of goals in most cases could increase the happiness of primary school teachers (Olčar et al., 2017). Spiritual intelligence emphasizes the ability to use spiritual resources. For example, primary school teachers with high spiritual intelligence have better mental models. They often look at problems from a higher spiritual level, take all things into consideration, and choose appropriate strategies to deal with daily problems. Unsurprisingly, they also have higher life satisfaction.

### The Moderating Role of Han and Ethnic Minorities

This study found that ethnic attributes have a moderating effect on the relation between awe and life satisfaction among primary school teachers in the southwestern minority areas of China. With an increase in awe level, the life satisfaction of minority teachers clearly increases, while the life satisfaction of Han teachers does not change noticeably.

According to the modernization theory, as the economy of a society develops, social, cultural, and psychological aspects of this society will also change. A strong economy can enhance the sense of self-efficacy and independence of people, leading to the emergence of a culture of individualism (Xu et al., 2016). These changes can weaken the sense of hierarchy in a collectivist culture, but the original culture of ethnic minorities remains relatively unaffected by modernization (Yin, 2005). Most of the ethnic settlements are in villages and towns, and the hierarchy in ethnic communities is significantly higher than in urban populations. A study by Liu and Wuyuntena. (2017) found that ethnic minorities emphasize both hierarchy and orderly equality based on the principles of matching resources, responsibilities, and power. These results show that individuals identify with the hierarchical structure of the group, which is a function of cultural adaptation. In this study, the majority of primary school teachers worked in villages and towns (i.e., accounting for 93.1% of the sample size), and many teachers worked in schools located in their hometowns. Research indicates that minority teachers are potentially influenced by their source culture. For example, the clothes of Yi peoples are mostly black, with the colors of black and white, respectively, connoting respect and inferiority (Xie et al., 2008). From a sociological perspective, social class and cultural values influence the awe experience of people to some extent. When a culture places more emphasis on social hierarchy, the experience of awe is more frequent and the level of awe is higher (Keltner and Haidt, 2003). Previous research indicated that “social class underlies differential patterns of attending to the self vs. orienting to others, lower social class was associated with more other-oriented feelings of compassion and love, and with greater awe” (Piff and Moskowitz, 2018). As mentioned earlier, these prosocial orientations help ethnic minority teachers to obtain more psychological satisfaction.

From cultural relativism, because of cultural diversity, people evaluate individual social success according to different values. Therefore, culture may treat life satisfaction as a relative concept (Diener and Suh, 2000). To some extent, awe partly reflects the core values of a culture, such as beliefs and codes of conduct. Ethnic minorities are concentrated in villages and towns, and traditional ethnic culture is inherently orderly. An ethnic group can maintain common recognition and clear values to a greater extent than non-ethnic groups. Compared with the Han, many awe triggers, such as natural deities, religious beliefs, and social rituals, may be more prevalent in ethnic minorities. This means that the awe experience of ethnic minority teachers was aroused more easily or more frequently in their daily life, which also means higher life satisfaction and happiness. Studies have shown that religious belief has a positive effect on the happiness of people (Purti, 2009; Zhang, 2009). For example, Zhang (2009) argued that the belief of Bimo, as a religious belief of Yi people, has a positive psychological integration effect on the subjective wellbeing of Yi people. The ceremony of Bimo takes place at least three times a year in a common family so that individuals often put themselves in the gathering of relatives and neighbors and get a sense of belonging and security in equal and intimate activities. In conclusion, compared to the Han teachers, awe derived from ethnic culture can better explain the life satisfaction of ethnic minority teachers and the difference of life satisfaction between ethnic minorities and Han among primary school teachers in previous studies.

### Limitations

First, the sample representativeness of ethnic minority teachers to reflect ethnic minority was insufficient. Single profession leads to a small external ecological validity and limitations in the generalization of these results. Therefore, the range of occupations and the diversity of the sample could be increased in future studies. Second, experimental research and intervention research should be combined in the future to further explore the possible causal relationship among awe, awe education, physical and mental health, and wellbeing in teachers.

## Conclusion

This study yields three key findings as follows:

(1) Awe, spiritual intelligence, and the life satisfaction of primary school teachers in the southwestern minority areas of China show a positive correlation with each other.
(2) Awe can indirectly affect life satisfaction through spiritual intelligence, which plays a partial mediating role in the relation between them.
(3) The ethnic attributes of primary school teachers had a moderating effect on the relation between awe and life satisfaction.

The results of this study imply that the inclusion of awe elements in teacher education, the construction of better spiritual resources, and a more optimized mental model can help teachers to deal intelligently with daily life and work problems and improve their happiness and life satisfaction. These implications suggest that this study has both psychological significance and practical value.

## Data Availability Statement

The raw data supporting the conclusions of this article will be made available by the authors, without undue reservation.

## Ethics Statement

The studies involving human participants were reviewed and approved by the Ethics Committee of Honghe University (Yunnan Province) in China. The patients/participants provided their written informed consent to participate in this study.

## Author Contributions

ZL was the primary investigator of the study, provided comments and ideas, and wrote the manuscript. TW and QX helped designed the study. XL and TJ provided comments and ideas and helped polished the writing of the article. All authors contributed to the article and approved the submitted version.

## Conflict of Interest

The authors declare that the research was conducted in the absence of any commercial or financial relationships that could be construed as a potential conflict of interest.
